# Biofortification of rice with the essential amino acid lysine: molecular characterization, nutritional evaluation, and field performance

**DOI:** 10.1093/jxb/erw209

**Published:** 2016-06-01

**Authors:** Qing-qing Yang, Chang-quan Zhang, Man-ling Chan, Dong-sheng Zhao, Jin-zhu Chen, Qing Wang, Qian-feng Li, Heng-xiu Yu, Ming-hong Gu, Samuel Sai-ming Sun, Qiao-quan Liu

**Affiliations:** ^1^Key Laboratory of Crop Genetics and Physiology of Jiangsu Province / Key Laboratory of Plant Functional Genomics of the Ministry of Education, College of Agriculture, Yangzhou University, Yangzhou, China; ^2^State Key Laboratory of Agrobiotechnology, School of Life Sciences, The Chinese University of Hong Kong, Shatin, Hong Kong, China; ^3^Co-Innovation Center for Modern Production Technology of Grain Crops of Jiangsu Province / Joint International Research Laboratory of Agriculture and Agri-Product Safety of the Ministry of Education, Yangzhou University, Yangzhou, China

**Keywords:** Field performance, grain quality, lysine, metabolic engineering, pyramid transgenic line, rice (*Oryza sativa* L.).

## Abstract

Rice (*Oryza sativa* L.), a major staple crop worldwide, has limited levels of the essential amino acid lysine. We previously produced engineered rice with increased lysine content by expressing bacterial aspartate kinase and dihydrodipicolinate synthase and inhibiting rice lysine ketoglutarate reductase/saccharopine dehydrogenase activity. However, the grain quality, field performance, and integration patterns of the transgenes in these lysine-enriched lines remain unclear. In the present study, we selected several elite transgenic lines with endosperm-specific or constitutive regulation of the above key enzymes but lacking the selectable marker gene. All target transgenes were integrated into the intragenic region in the rice genome. Two pyramid transgenic lines (High Free Lysine; HFL1 and HFL2) with free lysine levels in seeds up to 25-fold that of wild type were obtained via a combination of the above two transgenic events. We observed a dramatic increase in total free amino acids and a slight increase in total protein content in both pyramid lines. Moreover, the general physicochemical properties were improved in pyramid transgenic rice, but the starch composition was not affected. Field trials indicated that the growth of HFL transgenic rice was normal, except for a slight difference in plant height and grain colour. Taken together, these findings will be useful for the potential commercialization of high-lysine transgenic rice.

## Highlight

Marker-free transgenic rice that over-accumulates the essential amino acid lysine has been generated by combining lines with endosperm-specific and constitutive engineering of lysine, and has been evaluated in the field.

## Introduction

More than 50% of the human population worldwide has no access to a healthy, comprehensive diet of fresh foods (Zhu *et al.*, 2007). Rice (*Oryza sativa* L.) is a major food crop, with approximately one-third of the world’s population relying on rice as a staple diet and as the sole source of nutrition (Kusano *et al.*, 2015). However, milled rice grains are a poor source of essential amino acids, which are essential for normal growth and metabolism and cannot be synthesized *de novo* by humans, especially lysine (Lys) and methionine (Met) (Lee *et al.*, 2003; Ufaz and Galili, 2008; Cohen *et al.*, 2014). Therefore, increasing the lysine content in cereal grains, especially rice, is one of the main goals of breeders to enhance the nutritional value of grains and prevent nutrient-deficiency diseases, such as kwashiorkor (Toride, 2004).

Enhancing lysine content has been achieved in some cereals by a combination of conventional breeding and mutagenesis. For example, in a maize (*Zea mays* L.) line with the *opaque-2* mutation, the lysine content is up to 69% greater than that of wild-type maize (Mertz *et al.*, 1964; Nelson *et al.*, 1965). However, it would take a considerable amount of time, effort, and money to introgress the high-lysine trait into elite cultivars via conventional breeding. Moreover, due to the limited availability of lysine-rich germplasm resources, it is extremely difficult to improve this trait in most cereals, particularly rice (Sun and Liu, 2004). With the development of molecular biological techniques, three strategies have been developed to increase lysine levels in crops (Birla *et al.*, 2015). The direct approach involves expressing recombinant storage proteins with abundant lysine profiles, such as overexpressing lysine-rich proteins, in grains of rice (Wong *et al.*, 2015), maize (Yu *et al.*, 2004), and sorghum (*Sorghum bicolor* L.) (Zhao *et al.*, 2003). The second approach is to modify seed storage proteins. For example, silencing the gene encoding the 13-kDa prolamin increased total lysine content by 56% and altered nutritional quality in rice (Kawakatsu *et al.*, 2010). The third approach is to use metabolic engineering, which has been used to regulate the key genes involved in lysine metabolism to increase lysine content in plants (Zhu and Galili, 2003; Long *et al.*, 2013).

In higher plants, lysine, threonine (Thr), and Met are synthesized via the aspartate (Asp) family pathway, which is highly branched and regulated by a complex feedback mechanism (Galili *et al.*, 2005; Azevedo and Arruda, 2010). The lysine biosynthesis branch contains two key enzymes, aspartate kinase (AK) and dihydrodipicolinate synthase (DHPS) (Galili, 2002). AK functions in the first step of this pathway and is inhibited by both lysine and Thr, while DHPS functions in the lysine-specific branch and is inhibited by lysine (Galili *et al.*, 2016). Several studies have demonstrated the feasibility of increasing lysine levels by expressing mutated *AK* and/or mutated *DHPS*, which are insensitive to lysine feedback inhibition (Galili and Hoefgen, 2002; Qi *et al.*, 2011; Frizzi *et al.*, 2008, Long *et al.*, 2013). Simultaneously, another key enzyme, lysine ketoglutarate reductase/saccharopine dehydrogenase (LKR/SDH), which is involved in lysine catabolism, could be induced by the accumulation of lysine to balance amino acid flow (Zhu and Galili, 2003; Sun and Liu, 2004; Stepansky *et al.*, 2006). Thus, inhibiting or downregulating LKR/SDH enzyme activity represents another important strategy for improving lysine levels in plants (Hournard *et al.*, 2007; Reyes *et al.*, 2009). Moreover, the free lysine content of *Arabidopsis thaliana* seeds was dramatically increased by expressing a bacterial, feedback-insensitive *DHPS* gene while inhibiting the lysine catabolism pathway (Zhu and Galili, 2003). Similarly, the expression of bacterial feedback-insensitive *DHPS* together with an *LKR*/*SDH* RNAi construct also significantly increased free lysine contents in maize (Frizzi *et al.*, 2008). The use of a similar approach, that is, expressing the maize *DHPS* gene and inhibiting *LKR/SDH*, increased lysine levels in rice, but the free lysine level in mature rice seeds was only 4.0-fold higher than that of wild type (Lee *et al.*, 2001).

Biofortified crops exhibit some additional effects on various traits. For example, vegetative growth and seed development are affected in high-lysine transgenic tobacco (*Nicotiana tabacum*; Shaul and Galili, 1992), and the germination rates are quite slow in seeds from high-free-lysine transgenic canola (*Brassica campestris* L.), soybean (*Glycine max*), and Arabidopsis (Falco *et al.* 1995; Galili and Amir, 2013). In addition, the expression of a number of starch biosynthesis genes is altered by the maize *opaque2* mutation, which is associated with highly crystalline starch and contributes to the generation of a soft, starchy endosperm and improved protein quality (Jia *et al.*, 2013). Moreover, due to the random insertion of T-DNA in the genomes of transgenic plants, transgene expression levels are often affected by the location and structure of the T-DNA insertion (Oltmanns *et al.*, 2010; Inagaki *et al.*, 2015). Therefore, understanding the molecular structure of a transgenic locus is also important for future breeding programmes and subsequent commercialization (Głowacka *et al.*, 2016).

In a previous study, we generated various types of transgenic rice plants by expressing bacterial *AK* and *DHPS* and/or downregulating *LKR/SDH*, leading to the successful production of rice lines with increased lysine levels (Long *et al.*, 2013). Metabolic profiling of both seeds and leaves of these engineered rice plants suggested that the regulation of free lysine accumulation might differ between source and sink tissues (Long *et al.*, 2013). Therefore, in this study, with the aim of breeding transgenic rice with elevated lysine levels but lacking the selectable marker gene, we investigated the use of a combination of seed-specific and constitutive engineering of lysine metabolism by pyramiding two different transgenic events. Two pyramid transgenic rice lines with very high free lysine contents, named HFL1 and HFL2, were obtained. We evaluated the molecular characteristics, grain quality properties, and field performance of these selectable marker-free (SMF) transgenic rice lines compared to wild type. The results of this study lay the foundation for commercializing high-lysine transgenic rice.

## Materials and methods

### Plant materials, transgene constructs, and rice transformation

**Fig. 1.**
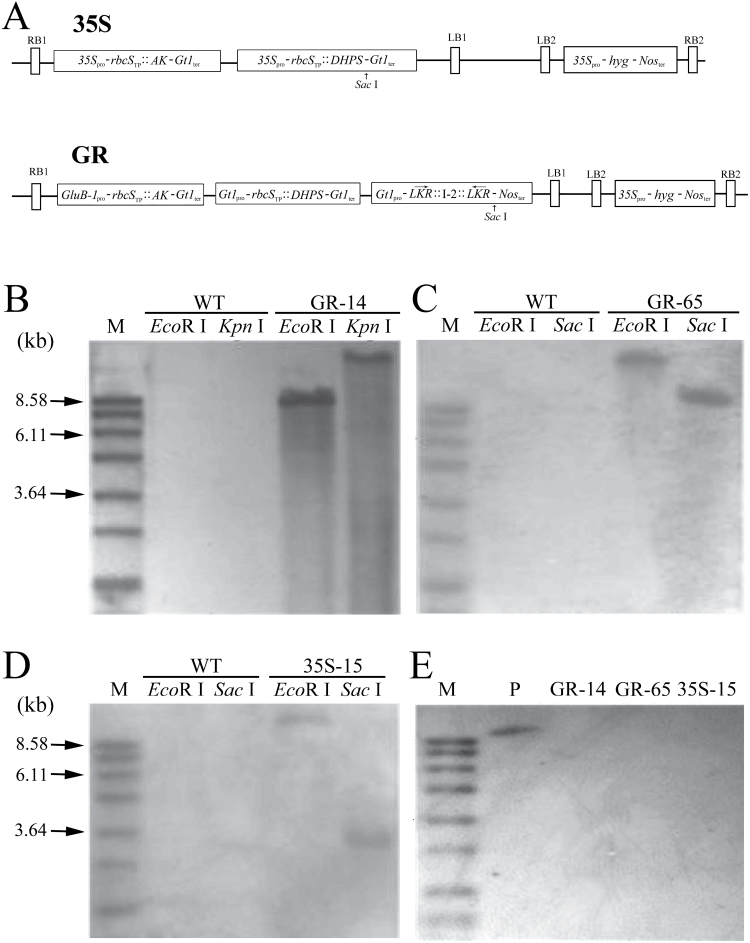
Rice transformation constructs and Southern blot analysis of genomic DNA from transgenic rice lines. **A** Structure of transgenic constructs 35S and GR. *AK* and *DHPS*, coding sequences of mutated *lysC* and *dapA*, respectively; *35S*_*pro*_, *CaMV 35S* promoter; *rbcS*_*TP*_, coding sequence of the chloroplast-targeting sequence of pea *rbcS*; *Gt1*_*pro*_ and *GluB-1*_*pro*_, rice glutelin *Gt1* or *GluB-1* promoter; *LKR*, partial sequence of rice *LKR/SDH* cDNA, with arrows showing the direction of gene transcription; and *I-2*, second intron of the rice glutelin gene *Gt1*. **B–E** Southern blot analysis of genomic DNA using the *AK* (B–D) or *Hyg* (E) probe. Total DNA was digested with *Eco* RI or other restriction enzymes as indicated (B–D). M, marker; P, GR plasmid DNA.

An elite *japonica* rice (*Oryza sativa* L.) cultivar, Wuxiangjing 9 (WXJ9, also indicated as wild type or WT), from China was used for transformation. Two transgene constructs (Fig. 1A), GR and 35S, were used to modify lysine biosynthesis and catabolism in rice. The construct 35S provides constitutive expression of the bacterial lysine feedback-insensitive *AK* (*lysC*) and *DHPS* (*dapA*) genes, both driven by the *CaMV 35S* promoter. The GR vector provides overexpression of these two bacterial genes, as well as downregulation of the rice endogenous *LKR/SDH* gene, with the three cassettes driven by the rice endosperm-specific glutelin *Gt1* or *GluB-1* promoter. The *lysC* and *dapA* genes were kindly provided by Professor Gad Galili, Weizmann Institute of Science, Israel (Shaul and Galili, 1992; Richaud *et al.*, 1986). Information about the three target genes and the chimeric constructs, rice transformation techniques, and molecular identification was presented in a previous report (Long *et al.*, 2013).

Because both chimeric constructs were cloned into the binary vector pSB130 with double T-DNA regions (Fig. 1A), one containing the above target transgenes (target T-DNA) and the other containing the hygromycin resistance gene (*Hyg*) as the selectable marker in the same construct, transgenic plants carrying only the target T-DNA could be separated from among the selfed progeny of some co-transformants. Thus, three elite SMF transgenic rice lines—35S-15 (harbouring the target T-DNA from the 35S construct) and both GR-14 and GR-65 (harbouring the target T-DNA from the GR construct)—were isolated and used for further experiments.

### Pyramiding transgenes by conventional crossing

**Fig. 2.**
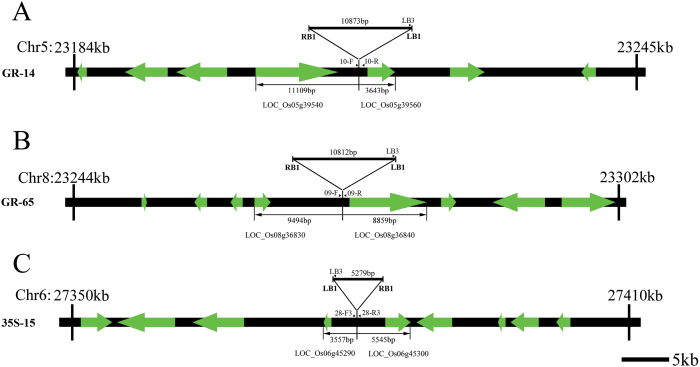
Schematic diagram of target T-DNA integration in the genomes of three transgenic rice lines. **A** T-DNA insertion site analysis of GR-14. **B** T-DNA insertion site analysis of GR-65. **C** T-DNA insertion site analysis of 35S-15. The thick lines between RB1 and LB1 represent the target T-DNA integrated in the rice genome; arrows show the predicted rice genes flanking the integrated T-DNA. The specific primers are indicated with small arrows. All analyses were based on the Nipponbare reference sequences.

Conventional crossing was carried out to combine the different transgenic events. The transgenic line 35S-15 was crossed with GR-14 or GR-65. Two pyramid lines, HFL1 (35S-15/GR-14) and HFL2 (35S-15/GR-65), were selected from the two crosses. During crossing and subsequent selection, the specific primers (see Supplementary Table S1 at *JXB* online, Fig. 2) for the above three transgenic lines were used to identify the target transgenes in lines HFL1 and HFL2. Selected homozygous transgenic lines in the F_2_ or later generations and the corresponding wild-type line were propagated for field analyses. All of the rice materials were planted in the greenhouse or paddy field at Yangzhou University (Yangzhou, Jiangsu Province, China).

### PCR and Southern blotting

Total genomic DNA was extracted from rice leaves as previously described (Murray and Thompson, 1980). The primers used for PCR are listed in Supplementary Table S1 and the locations of some specific primers are indicated in Fig. 2. For Southern blot analysis, aliquots of total DNA were digested with the suitable restriction endonucleases, separated on an agarose gel, and transferred to Hybond-N+nylon membranes (Roche). A fragment of the bacterial *AK* or *Hyg* gene was labelled with digoxigenin (DIG) using a DIG nucleic acid labelling kit (Roche) and used as a hybridization probe. Hybridization, washing, and signal detection were carried out using a DIG Luminescent Detection Kit (Roche) following the manufacturer’s instructions.

### Isolation of T-DNA flanking sequences

The flanking sequences of the integrated target T-DNA in the rice genome were isolated using inverse-PCR combined with thermal asymmetric interlaced PCR (Liu and Huang, 1998), with some modifications. In brief, the total genomic DNA from transgenic rice plants was digested with the restriction endonuclease *Sac*I and ligated using T_4_ DNA ligase. After purification, the ligated total DNA was used as a template for three rounds of PCR with three pairs of nested primers (SP1–SP6; Supplementary Table S1). The nested primers were designed according to sequences of the target T-DNA in vector GR or 35S and the sequence located near the first left border (LB1; SP1–SP3) and *Sac*I site (SP4–SP6), respectively. The PCR products from the final round of amplification were cloned and sequenced. The verified sequences were aligned using tools from the NCBI website and compared with known T-DNA sequences.

### Quantitative RT-PCR and western blot analysis

Total RNA was extracted from developing rice seeds at 15 days after flowering (DAF) using the cold-phenol method. The isolated RNA was purified and reverse transcribed into cDNA for quantitative reverse-transcription PCR (qRT-PCR) analysis. The primers are listed in Supplementary Table S1, and the rice *actin* gene was used as an internal control. Total seed proteins were extracted from developing seeds as described (Long *et al.*, 2013). After separation by SDS-PAGE, proteins were detected through western blotting using antibodies specific for bacterial AK and DHPS, or rice AK, DHPS, and LKR.

### Field trial and analysis of agronomic traits

The five selected transgenic rice lines and their wild type were grown in the experimental field in Yangzhou, Jiangsu Province, China, with permission for small-scale field trials from the Ministry of Agriculture, China. Three repeated plots were used, and rice lines were planted randomly in each plot. The main agronomic traits were recorded after maturity, and the mature seeds were harvested for quality analysis and feeding experiments. Rice preparation and subsequent general quality measurements, including apparent amylose content (AAC), gel consistency (GC), Rapid Visco Analyser (RVA) profile, and starch and protein contents, were performed according to a previous report (Zhu *et al.*, 2010).

### Amino acid analysis

For free amino acid (FAA) analysis, 50mg of milled and dried rice power was thoroughly mixed in 500 μL Na-S™ buffer (2% sodium citrate, 1% HCl, 0.1% benzoic acid; Beckman, USA) for 30min in a mixer and extracted for 10min via ultrasonication. The mixture was then centrifuged at 16 000rpm for 10min at room temperature. The supernatant was collected and filtered through a 0.45-μm nylon membrane syringe filter (Pall Life Sciences, USA) for injection and analysis using an L8900 Amino Acid Analyzer (Hitachi, Japan).

For total amino acid (TAA) analysis, 10mg of dry rice power was hydrolysed with 1mL of 6 N HCl (Sigma, USA) in a 2-mL screw-cap tube, followed by the addition of 10 nmol L(+)-norleucine (Wako Pure Chemicals, Japan). The samples were heated at 110°C for 24h, and the HCl was then evaporated for 6h at 65°C. Dried samples were dissolved in 1mL Na-S™ buffer and filtered for amino acid analysis as described above. Data obtained from HPLC were normalized with the level of L(+)-norleucine per sample. For each sample, two technical replicates were carried out.

### Statistical analysis

For sample characterization, at least three replicate measurements were taken, unless otherwise specified. All data were reported as the mean ± SD. The results were analysed via ANOVA (SPSS version 13.0), and the differences in these characters between the transgenic samples and the wild type were analysed.

## Results

### Production of SMF GR and 35S transgenic lines

To produce rice that accumulates lysine in the endosperm by enhancing lysine biosynthesis and blocking its catabolism, we generated the GR vector in a twin T-DNA vector backbone (Fig. 1A), in which one T-DNA (target T-DNA) contained the bacterial lysine feedback-insensitive *AK* and *DHPS* genes as well as the *LKR*-RNAi construct, all driven by the rice endosperm-specific *glutelin* promoter. Using *Agrobacterium*-mediated transformation, over 100 independent transgenic lines were obtained in the background of the elite *japonica* cultivar WXJ9. Among the progenies of these original transgenic plants, two independent transformants, designated GR-14 and GR-65, were isolated that contained the target T-DNA with the three target transgenes (*AK*, *DHPS*, and *LKR*-RNAi; Fig. 1B, C) but lacked T-DNA with the selectable marker gene (*Hyg*; Fig. 1E). Only one band was detected by Southern blot analysis when the genomic DNA from GR-14 or GR-65 was digested with different restriction endonucleases (Fig. 1B, C), implying that both GR transgenic lines contained a single copy of the target T-DNA.

In a previous study (Long *et al.*, 2013), we generated several elite 35S transgenic lines in the background of the same cultivar, WXJ9, with constitutive expression of both bacterial *AK* and *DHPS* driven by the *CaMV 35S* promoter (Fig. 1A). Among these transgenic lines, the 35S-15 transgenic line also contained only one copy of the target T-DNA with the bacterial *AK* and *DHPS* (Fig. 1D) but not the T-DNA with the *Hyg* selectable marker gene (Fig. 1E). Therefore, three SMF transformants with a single copy of the target T-DNA in their genomes—35S-15, GR-14, and GR-65—were obtained from selfed progenies and used for further molecular, field, and quality evaluations.

### The target T-DNA is integrated within the intragenic region of the rice genome

To determine the integration sites of the target transgenes in the selected SMF transgenic lines, we isolated the flanking sequences of the target T-DNA in the rice genome using inverse-PCR with some modifications. After three rounds of PCR amplification, one specific product was obtained for each transformant. After sequencing and alignment with known reference sequences, the junction and flanking sequences were successfully identified in all three SMF transgenic lines (Fig. 2, and see Supplementary Fig. S1 at *JXB* online). In line GR-14, the target T-DNA was inserted near nucleotide 23 216kb on chromosome 5 (based on the Nipponbare reference sequence), which is located between LOC_Os05g39540 and LOC_Os05g39560 (Fig. 2A). In the other GR line (GR-65), the target transgenes were located between LOC_Os08g36830 and LOC_Os08g36840 on chromosome 8 (Fig. 2B). In line 35S-15, the target T-DNA was integrated near nucleotide 27 381kb on chromosome 6, which is located within the intragenic region between two predicted genes, LOC_Os06g45290 and LOC_Os06g45300 (Fig. 2C).

Sequence alignment and analysis showed that all of the integration sites of the three target T-DNAs were located within the intragenic regions of the transgenic rice genomes. Moreover, we analysed the junction sequences between the T-DNA and rice genomes, finding that there were always several nucleotide changes within the junction sites (see Supplementary Fig. S1 at *JXB* online), but all of the target transgenes (including the promoter and terminator) were completely integrated in the genome.

### Free lysine content increased in the GR transgenic lines but not the 35S-15 transgenic line

**Fig. 3.**
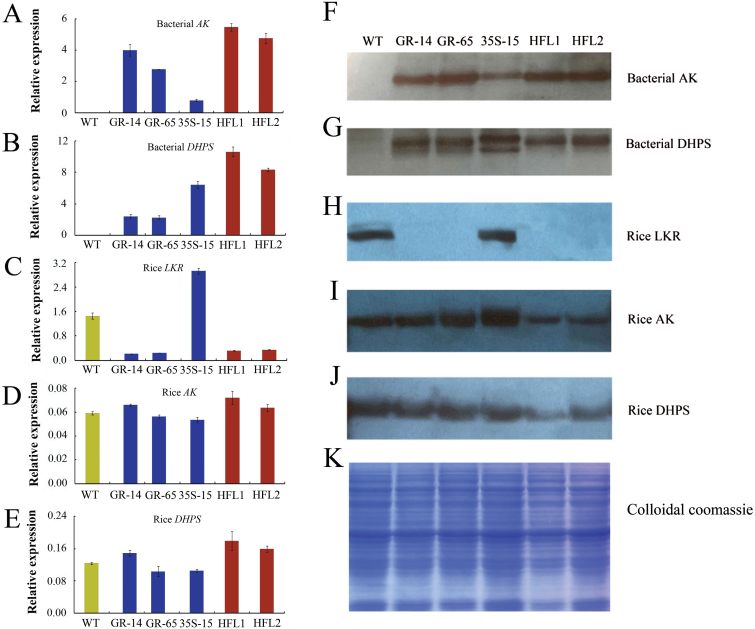
Expression analysis of *AK*, *DHPS*, and *LKR/SDH* in developing rice seeds. **A–E** Quantitative RT-PCR analysis of bacterial *AK* and *DHPS* and rice *AK*, *DHPS*, and *LKR* transcript levels in developing seeds at 15 DAF in WT and five transgenic lines. **F–J** Western blot analysis of bacterial AK and DHPS and rice AK, DHPS, and LKR protein extracts from developing seeds at 15 DAF. **K** SDS-PAGE analysis of total proteins. Equal protein amounts were loaded according to Coomassie brilliant blue stained gels.

**Fig. 4.**
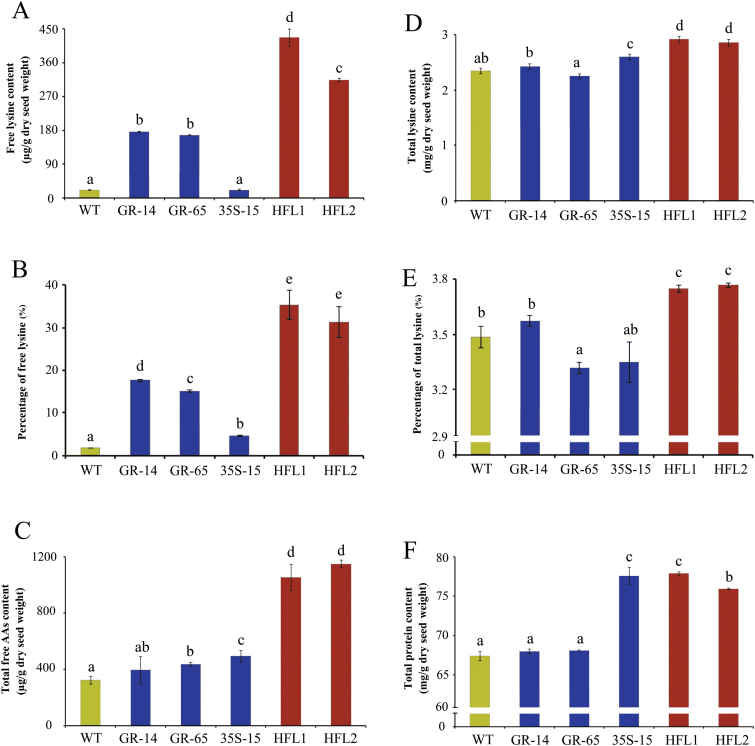
The contents of lysine, total amino acids and proteins in mature seeds of transgenic and wild-type rice. **A** Free lysine content (μg/g dry seed weight). **B** Percentage of total lysine per total free amino acids (in weight). **C** Total free amino acid content (μg/g dry seed weight). **D** Total lysine content (mg/g dry seed weight). **E** Percentage of total lysine per total proteins (in weight). **F** Total protein content (mg/g dry seed weight). Error bars represent SDs of three biological replicates, and the different letters indicate statistical significance among transgenic plants and wild type at *P* < 0.05.

Both qRT-PCR and western blot analyses showed that the bacterial *AK* and *DHPS* genes were highly expressed in developing seeds at 15 DAF in the two GR transgenic lines, but not in the wild type (Fig. 3A, B, F, G). In these GR lines, the expression of the endogenous rice *LKR/SDH* gene was inhibited in developing seeds, as expected (Fig. 3C, H). Moreover, the expression of bacterial *AK* and *DHPS* was not detected in other vegetative tissues of the GR lines, such as leaves (see Supplementary Fig. S2 at *JXB* online), indicating the endosperm-specific expression of the introduced genes in the selected transformants. The levels of free lysine in transgenic lines GR-14 and GR-65 were significantly increased compared to wild type, with levels 10.1-fold and 9.6-fold more than that of wild type, respectively (Fig. 4A).

In the 35S-15 transgenic line, the bacterial *AK* and *DHPS* genes were constitutively expressed in most tissues, including developing seeds (Fig. 3A, B, F, G) and leaves (see Supplementary Fig. S2 at *JXB* online). However, the free lysine content in the seeds of 35S-15 transgenic plants did not differ from that of wild type (Fig. 4A), perhaps because the overexpression of bacterial *AK* and *DHPS* may induce lysine degradation (Long *et al.*, 2013). As expected, the expression of the endogenous rice gene *LKR/SDH* was much higher in the developing seeds of 35S-15 plants (approximately 5-fold) than in wild type (Fig. 3C, H).

### Pyramiding of 35S and GR transgenes

**Fig. 5.**
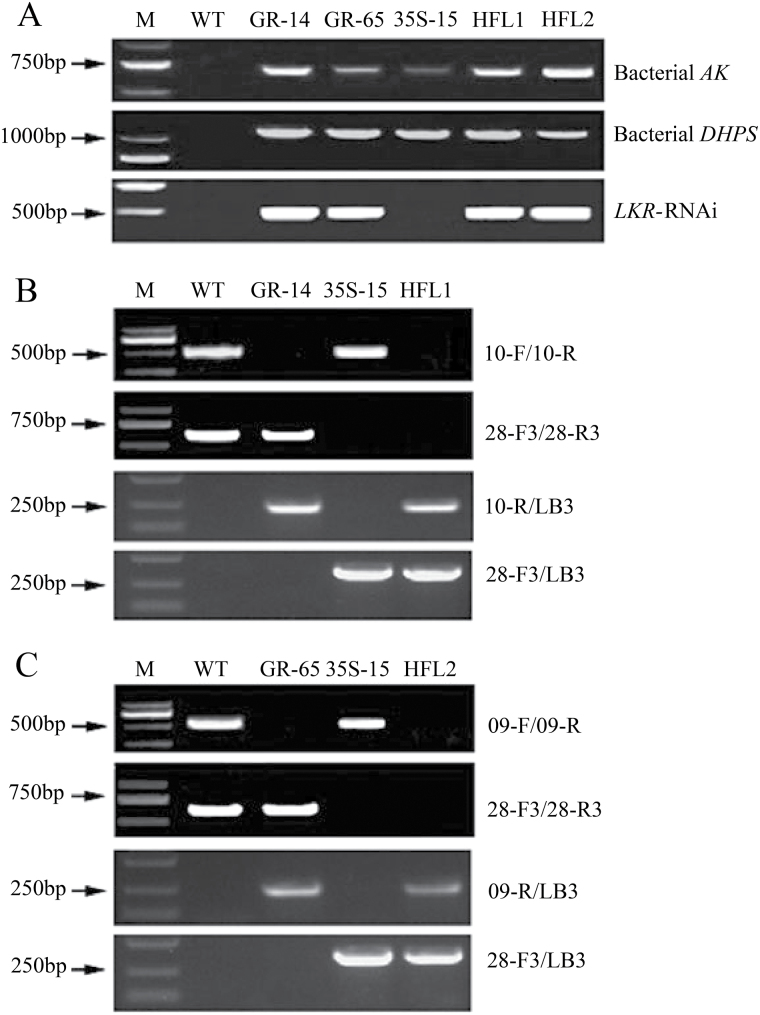
PCR identification of pyramid transgenic lines HFL1 and HFL2. **A** PCR analyses of bacterial *AK* and *DHPS* transgenes and rice *LKR*-RNAi construct. **B** PCR confirmation of 35S-15, GR-14, and combined line HFL1 using specific primers, as indicated on the right. **C** PCR confirmation of 35S-15, GR-65, and combined line HFL2 using specific primers, as indicated on the right. The locations of the primers are shown in Fig. 2.

Because the expression patterns of the bacterial *AK* and *DHPS* genes differed in the GR and 35S transgenic lines, we investigated whether more lysine would accumulate in the endosperm by pyramiding these two transgenic events. Thus, the 35S-15 transgenic plant was crossed with GR-14 and GR-65, respectively, and two pyramid transgenic lines, designated HFL1 and HFL2, were selected from these crosses. During crossing and subsequent selection, specific primers for the above three target transgenes were used to identify lines HFL1 and HFL2 (Fig. 5A).

We also utilized several sets of specific primers designed based on the target T-DNAs and their flanking regions in the rice genome to further confirm these two combined lines (Fig. 2, Fig. 5B, C). For example, as shown in Fig. 5B, when PCR was performed on pyramid line HFL1 from the cross between 35S-15 and GR-14 using primers 10-F and 10-R, which are located within the flanking region on both sides of the target T-DNA in GR-14 (Fig. 2A), the expected amplification product was only detected in wild type and 35S-15 due to the lack of an insert between these two primer sequences, but not in the homozygous transgenic line GR-14 or its derivative HFL1 (Fig. 5B). However, when we used primers LB3 and 10-R, which are located in the left border of the target T-DNA in the GR vector and its flanking region, respectively, in GR-14 (Fig. 2A), no product was amplified in wild type or 35S-15, whereas the expected product was produced in homozygous transgenic plant GR-14 and combined line HFL1 (Fig. 5B). Thus, two new SMF pyramid transgenic lines, HFL1 and HFL2, were selected and found to contain twin transgenic events, as expected based on their 35S and GR parents.

In developing seeds of the pyramid transgenic rice HFL1 and HFL2, the expression of both bacterial *AK* and *DHPS* was much higher than that of the 35S and GR parents (Fig. 3A, B, F, G). Meanwhile, the expression level of rice *LKR/SDH* was also reduced, as in the GR parents, but was significantly lower than that of the other parent, 35S-15. Thus, the pyramid transgenic rice lines exhibited the combined advantages of the two parents in terms of lysine metabolism. In addition, we also investigated the expression of endogenous rice *AK* and *DHPS* genes. As shown in Fig. 3, overexpressing bacterial *AK* and *DHPS* and downregulating *LKR* expression in transgenic rice had little or no effect on the expression of endogenous rice *AK* or *DHPS* (Fig. 3D, E).

### Accumulation of very high free lysine levels in pyramid transgenic rice

The pyramid transgenic plants grew normally, like the parents and wild type, in the greenhouse and field. We therefore harvested mature seeds from these lines for component analysis. Importantly, the free lysine contents in mature seeds were dramatically higher in pyramid lines HFL1 and HFL2 than in the GR and 35S parents (Fig. 4A), with values of 437.8 and 346.3 μg/g (dry weight) in seeds of HFL1 and HFL2, respectively. These values are 2.5-fold and 2.1-fold more than those of the GR parents, respectively, and approximately 25.3-fold and 19.7-fold more than that of wild-type line WXJ9.

Interestingly, the increase in free lysine levels in transgenic rice seeds was associated with a significant increase in the contents of all measurable FAAs, especially in HFL1 and HFL2, with levels 3.3-fold and 2.5-fold that of wild type, respectively (Fig. 4C). In addition, the percentage of free lysine per total measurable FAAs was 35.3% and 31.3% in HFL1 and HFL2, respectively, but only 2.6% in wild type (Fig. 4B). These results imply that the increased total measurable FAA levels in HFL1 and HFL2 were mainly due to the accumulation of free lysine in the endosperm.

Based on the elevation in free lysine content, we compared the total lysine content after protein hydrolysis between transgenic seeds and wild type. As shown in Fig. 4D, though there was an obvious increase in free lysine levels in mature seeds in both GR transgenic lines, there was little or no difference in total lysine contents in GR-14 and GR-65 compared to wild type, perhaps due to the low proportion of free lysine. In 35S-15, there was no increase in free lysine content, but there was a slight increase in total lysine content, which might have been due to the increase in TAA and total protein contents. In the pyramid transgenic lines, a large increase in total lysine content was detected in mature seeds compared to wild type and the GR and 35S parents. The total lysine content in mature seeds of HFL1 and HFL2 rice was 24% and 19% higher than that of wild type line WXJ9, respectively (Fig. 4D). The total protein content in mature seeds of the pyramid transgenic lines was 16% higher (HFL1) and 13% higher (HFL2) than that of wild type, and the percentage of lysine per total protein was much higher than that of the other lines (Fig. 4E, F).

Overall, the pyramid transgenic lines exhibited very high free lysine content as well as increased total lysine content in mature seeds. Moreover, we examined the lysine content among several successive generations (years) and found that the elevated lysine content was stably inherited (see Supplementary Fig. S3 at *JXB* online).

### The accumulation of other amino acids in the HFL pyramid lines

**Fig. 6.**
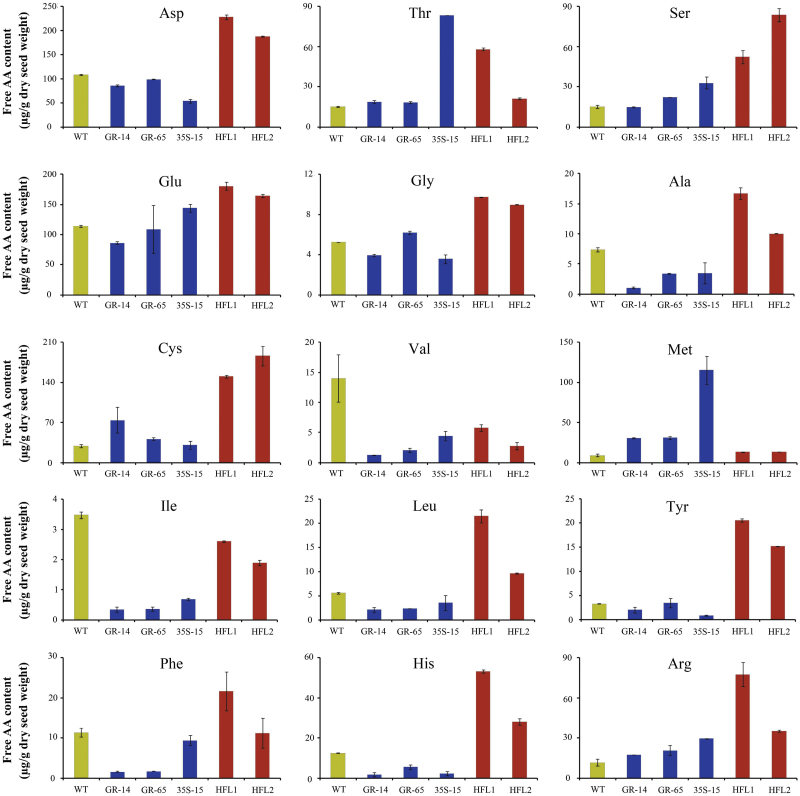
The contents of other measurable free amino acids in mature seeds of transgenic and wild-type rice. Data are presented as μg/g dry seed weight.

To examine the effects of engineered lysine metabolism on the accumulation of other amino acids in seeds, we compared each measurable amino acid level between the transgenic lines and wild type (Fig. 6). In addition to lysine, the levels of most FAAs in seeds (per dry weight) were also significantly elevated in the transgenic lines (Fig. 6). However, the percentage of each amino acid in the total measurable FAA pool was greatly reduced (see Supplementary Fig. S4 at *JXB* online). For example, although the true contents of glutamate (Glu), Asp, Thr, and Met were much higher in the pyramid lines than in wild type, their relative concentrations in the total FAA pool were significantly lower in the two HFL lines than in wild type. These results suggest that a complex regulatory network determines the overall levels of these amino acids, especially Glu, Asp, Thr, and Met, which are intermediates or products of the aspartate pathway. Overall, engineering of lysine metabolism in HFL transgenic plants led to high accumulation of most lysine-related amino acids, while lysine was dominant. Thus, high free lysine contents as well as high lysine levels in seeds were achieved in the pyramid transgenic rice lines.

### Field performance and general grain quality of HFL transgenic lines

During the growing season, the five SMF transgenic lines grew normally, like the wild type (see Supplementary Fig. S5 at *JXB* online). We investigated the main agronomic traits of the transgenic lines and the wild type after maturation (see Supplementary Fig. S6 at *JXB* online). Most traits were similar between the transgenic plants and wild type. The plant height of 35S-15 and the two pyramid lines (HFL1 and HFL2) was shorter than that of wild type (Supplementary Fig. S6). The panicle length of 35S-15 was also shorter than that of wild type, but normal in the pyramid transgenic rice. No difference in tiller, seed number, or grain weight was observed between the transgenic lines and wild type, suggesting that the selected SMF transgenic lines, including the two pyramid lines, could maintain yields as high as those of the wild-type line WXJ9, an elite, high-yielding *japonica* rice cultivar. Indeed, the two HFL pyramid rice lines exhibited comparable yields to that of wild type (Supplementary Fig. S6G).

**Fig. 7.**
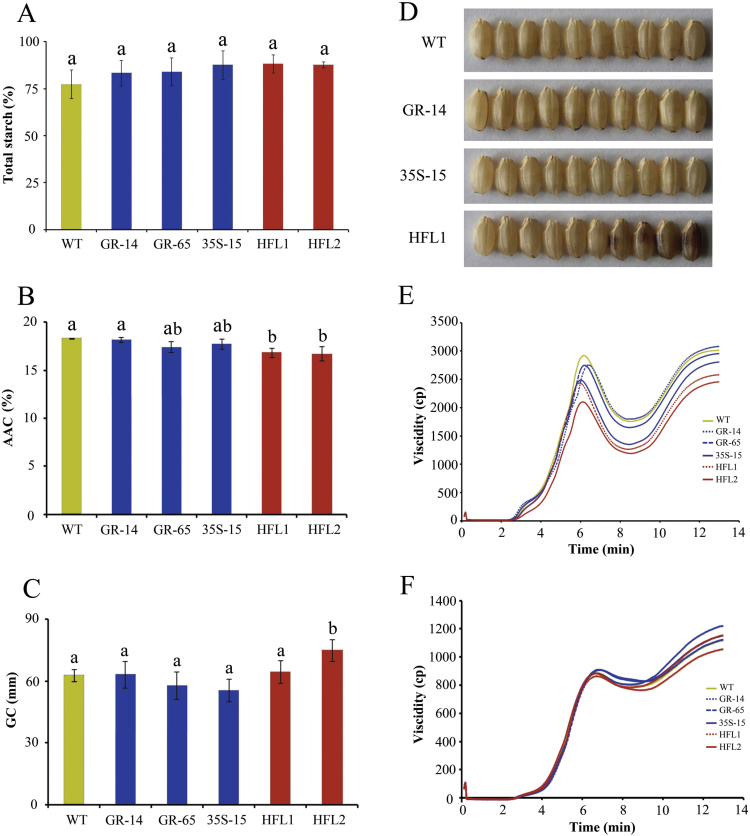
The physicochemical qualities and appearance of mature seeds from transgenic and wild-type rice. **A** Total starch content in rice flours (g/100g dry rice flour). **B** Percentage of apparent amylose content (AAC) in rice flours (g/100g dry rice flour). **C** Gel consistency (GC, mm) of rice flours. **D** Appearance of brown rice. **E, F** RVA spectra of rice flours (E) and starches (F). The error bars refer to SDs of three biological replicates. The different letters indicate statistical significance among transgenic plants and wild type at *P* < 0.05.

In terms of general grain quality, there was also no difference in chalkiness among rice lines, but some mature grains of the pyramid rice appeared dark brown (Fig. 7D). In addition, the total starch content in transgenic seeds was slightly but not significantly higher than that of wild type (Fig. 7A). The AAC was similar between GR, 35S, and wild type but was significantly lower in the two pyramid lines (Fig. 7B). The GC was not significantly different between the wild type and transgenic lines, except for HFL2, in which the GC was slightly softer than that of wild type (Fig. 7C).

We analysed the pasting properties of rice grains using an RVA, because pasting is one of the most important indexes used to evaluate rice cooking and eating quality (Chun *et al.*, 2015). First, the rice flours were subjected to RVA analysis (Fig. 7E). The RVA curves were identical between the two GR transgenic lines and the wild type, but the RVA values were much lower in the three other transgenic lines, especially the two pyramid lines, than in wild type. Several parameters of the RVA profile, such as peak viscosity, hot paste viscosity, and cool paste viscosity, were reduced in HFL1 and HFL2 flours (see Supplementary Table S2 at *JXB* online). We also measured the pasting property of purified starches (Fig. 7F), finding that the RVA curves were similar among all transgenic lines and wild type. Taken together, these results suggest that the engineering of lysine metabolism and the increase in free lysine content had no effect on starch content and properties, but it had a large impact on the physicochemical characteristics of rice flours, especially for the two pyramid lines with very high levels of free lysine.

## Discussion

Metabolic engineering is an effective approach for increasing lysine contents in the sink tissues of crops. In plants, lysine accumulation is controlled by not only its biosynthesis but also its degradation. Increasing lysine biosynthesis might trigger the degradation of more lysine, which limits the increase in final levels of free lysine (Falco *et al.*, 1995; Galili, 2002). In agreement with this, in the present study as well as our previous study (Long *et al.*, 2013), we only detected a slight or no increase in lysine content in the seeds of 35S-15 transgenic rice with constitutive expression of bacterial *AK* and *DHPS* (Fig. 4A). A more promising strategy for increasing lysine accumulation is to simultaneously manipulate both the lysine biosynthesis and catabolic pathways (Galili, 2002), a strategy that has been highly successful in Arabidopsis (Zhu and Galili, 2003) and in other crops (Lee *et al.*, 2001; Frizzi *et al.*, 2008).

In this study, we generated GR transgenic rice with both endosperm-specific expression of bacterial *AK* and *DHPS* and inhibition of rice *LKR/SDH* expression (Fig. 1A). In two selected SMF transgenic rice lines, the free lysine content was significantly higher than in wild type (Fig. 4A), with levels approximately 10-fold higher than those of wild type. The free lysine level was much lower than that in 35R transgenic rice harbouring the same *LKR*-RNAi construct but with constitutive expression of bacterial *AK* and *DHPS*, produced in our previous study (Long *et al.*, 2013). Therefore, we further introgressed the 35S transgenic event from 35S-15 transgenic rice into the GR background. We successfully obtained two pyramid transgenic rice lines with combined endosperm-specific and constitutive expression of two lysine biosynthesis genes, as well as suppression of *LKR/SDH*. As expected, the free lysine content was dramatically increased in the pyramid transgenic lines, with levels up to 25-fold of those in wild type. These results, combined with the results of our previous study (Long *et al.*, 2013), indicate that engineering of lysine metabolism in both the source and sink tissue is highly effective for accumulating high levels of free lysine in rice seeds.

In higher plants, lysine is synthesized from Asp via one of the branches of the Asp family pathway, while Glu is mainly synthesized from glutamine (Gln) and is also a product of the lysine catabolism pathway (Galili *et al.*, 2005). In the current study, the levels of free Asp and Glu increased somewhat in the pyramid transgenic rice, although their relative ratios per total measurable FAAs decreased (Fig. 6, Supplementary Fig. S4). Notably, the level of cysteine (Cys), the precursor for Met biosynthesis, was significantly increased in HFL1 and HFL2 (Fig. 6); this amino acid is also the precursor for glutathione biosynthesis and is associated with oxidative stress (Noctor *et al.*, 2012). By contrast, the levels of free Met, the downstream product of Cys, were markedly increased in 35S-15 seeds. Additionally, the relative levels of alanine (Ala), isoleucine (Ile), valine (Val), and leucine (Leu), which are derived from pyruvate, also decreased in transgenic rice seeds. The levels of the branched-chain amino acids Val and Leu were reduced in these plants, perhaps to maintain the functionally required homeostasis (Binder *et al.*, 2007).

In previous studies, obvious pleiotropic effects were usually detected in high-lysine transgenic plants, includes changes in seed phenotype, oil content, germination, and even yields (Shaul and Galili, 1992, 1993; Tzchori *et al.*, 1996; Zhu and Galili, 2003; Angelovici *et al.*, 2011). In addition, a broader analysis of metabolic pathways indicated that genes for lysine biosynthesis are generally downregulated under abiotic stress (Less *et al.*, 2011). In the present study, there was no difference in major agronomic traits and yield, except for the plant height of 35S-15 transgenic rice, between the transgenic lines and wild type based on field trials (Supplementary Fig. S6), indicating that the transgenes had no obvious adverse effects on rice morphology. The plant height was significantly lower in 35S-15 transgenic rice than in wild type, whereas no difference was found in GR transgenic lines versus wild type (Supplementary Fig. S6). The plant height was also slightly reduced in the two pyramid transgenic rice lines derived from the same 35S-15 line (Supplementary Fig. S6), suggesting that the constitutive expression of bacterial *AK* and *DHPS* under the control of the *CaMV 35S* promoter might affect plant growth. It is possible that *AK* expression is regulated by photosynthesis-related metabolites, such as sucrose and inorganic phosphate (Zhu-Shimoni *et al.*, 1998).

The high levels of free lysine in mature grains had a great impact on the physicochemical characteristics of milled rice, particularly the levels of total FAAs and proteins, in 35S-15 transgenic rice and the pyramid lines (Fig. 4C, F). These results indicate that constitutive engineering of lysine biosynthesis will lead to the biosynthesis of increased levels of relative amino acid and subsequent protein synthesis in rice endosperm. The increased amino acid and protein levels have an effect on rice cooking and eating quality (Lyon *et al.*, 2000; Tan and Corke, 2002). Our results show that the accumulation of lysine as well as other relative amino acids in both pyramid transgenic lines appears to promote better rice quality compared to wild type, as there was a slight decrease in amylose content and the pasting curve (Fig. 7). These results suggest that all of the components examined, including FAAs and proteins, ultimately contribute to rice texture, indicating that there is a complex regulatory mechanism in grains (Jia *et al.*, 2013; Gayral *et al.*, 2016). In addition to the physicochemical properties, we observed a large impact on the colour of brown rice in the two pyramid rice lines (Fig. 7D), which has not been reported in other species. This suggests that the lysine metabolic pathway might differ among species. Several studies have indicated that lysine metabolism from the Asp family pathway involves multiple metabolic pathways, including amino acid metabolism, cellular energy metabolism, and plant stress responses (Galili and Amir , 2013; Kiyota *et al.*, 2015). Therefore, engineering of lysine metabolism in the transgenic rice lines produced in the present study might have affected other metabolic pathways, leading to the seeds with a brown tint. Details about this colour change require further study.

When considering the potential commercialization of high-lysine rice, it is important to investigate the integration of target transgenes as well as the selectable marker gene (Jiang *et al.*, 2014; Bartlett *et al.*, 2014). In the current study, we elucidated the molecular characteristics of the insertion of the target genes into the genomes of both pyramid transgenic rice lines. In both pyramid lines as well as the 35S and GR parents, the *Hyg* selectable marker gene was eliminated, and the insertion sites of all target transgenes were not located in the genetic regions of endogenous genes. Therefore, it appears that integration of the transgene will have little or no impact on the expression of rice genes and gives an explicit statement for the potential safety assessment of selected transgenic rice with high free lysine in the future.

## Supplementary data

Supplementary data are available at *JXB* online.

**Figure S1.** Junction and flanking sequences of the target T-DNA in three transgenic lines.

**Figure S2.** Quantitative RT-PCR analysis of bacterial *AK* (A) and *DHPS* (B) transcript levels in leaves of wild-type and transgenic rice.

**Figure S3.** The contents of free (A) and total lysine (B) in mature grains of transgenic and wild-type rice.

**Figure S4.** Comparison of the percentage (by weight) of other individual free amino acids to total measurable free amino acids.

**Figure S5.** Growth performance of wild type and HFL1 rice in the field.

**Figure S6.** The major agronomic traits of transgenic and wild-type plants.

**Table S1.** Primers used in this study.

**Table S2.** Parameters of RVA profiles of flours and purified starches from mature rice grains.

Supplementary Data

## Abbreviations:

AAC: apparent amylose content
AK: aspartate kinase
DAF: days after flowering
DHPS: dihydrodipicolinate synthase
FAA: free amino acids
GC: gel cosistency
LKR/SDH: lysine ketoglutarate reductase/saccharopine dehydrogenase
HFL: high free lysine
Hyg: hygromycin resistance gene
RVA: Rapid Visco Analyser
SMF: selectable marker free;
TAA: total amino acids.
