# Supplementary material for: Biofortification of rice with the essential amino acid lysine: molecular characterization, nutritional evaluation, and field performance
Source: J Exp Bot. 2016 Jun 1;67(14):4285–96. doi: 10.1093/jxb/erw209 (PMC5301931; doi:10.1093/jxb/erw209)
Supplement: Supplementary Data [file supp_67_14_4285__index.html]

Biofortification of rice with the essential amino acid lysine: molecular characterization, nutritional evaluation, and field performance — Biofortification of rice with the essential amino acid lysine: molecular characterization, nutritional evaluation, and field performance — Supplementary Data 

# Biofortification of rice with the essential amino acid lysine: molecular characterization, nutritional evaluation, and field performance

## Supplementary Data

Data files

- supplementary\_figures\_S1\_S7\_Tables\_S1\_S2.pdf - Supplementary Data
