# Supplementary material for: Biofortification of rice with the essential amino acid lysine: molecular characterization, nutritional evaluation, and field performance
Source: J Exp Bot. 2016 Jun 1;67(14):4285–96. doi: 10.1093/jxb/erw209 (PMC5301931; doi:10.1093/jxb/erw209)
Supplement: Supplementary Data [file supp_erw209_supplementary_figures_S1_S7_Tables_S1_S2.pdf]

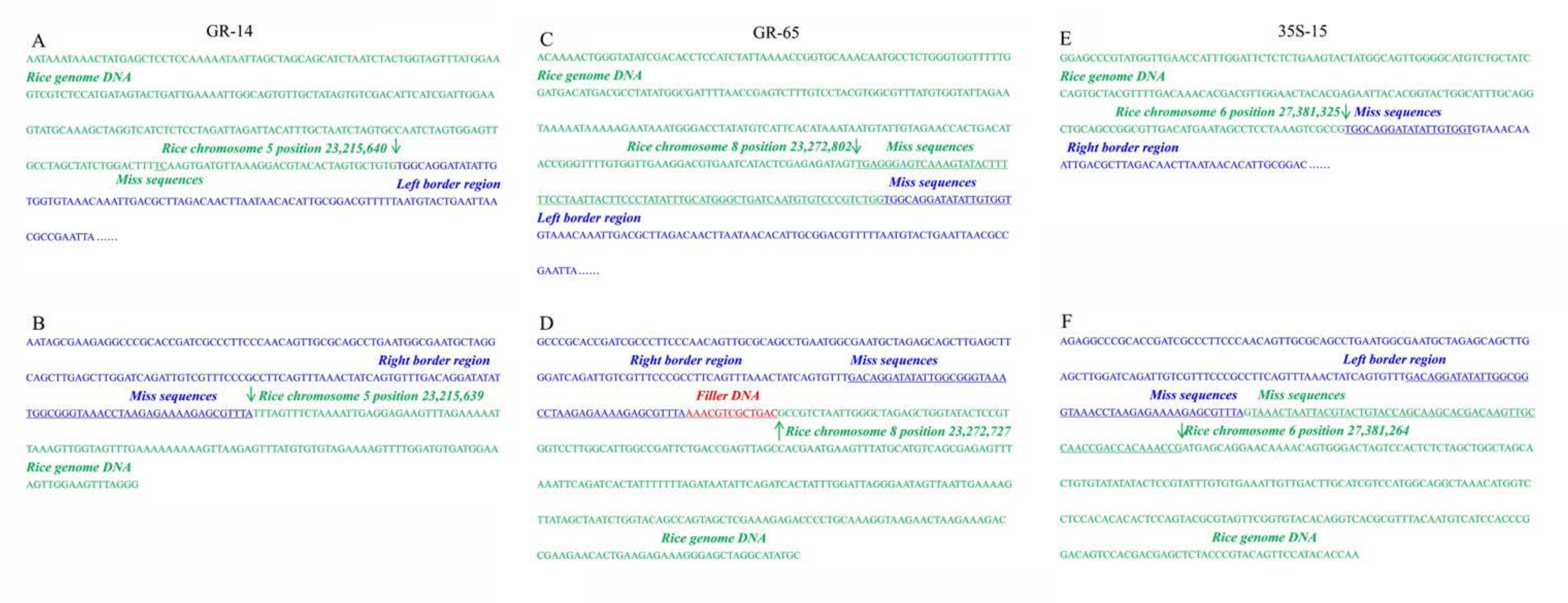

Supplementary Figure S1. Junction and flanking sequences of the target T-DNA in three transgenic lines. (A-B) GR-14, (C-D) GR-65, (E-F) 35S-15. A, C and E are left border regions and the flanking rice genome sequences. B, D and F are right border regions and the flanking rice genome sequences. Left and right border regions are presented in blue letters, and the miss sequences of them are presented in underline blue. Filler DNAs are depicted in underline red letters. Rice genome sequences are presented in green letters, and the miss sequence of them are presented in underline green. Arrows indicate the junction position based on rice genome (http://www.gramene.org/).

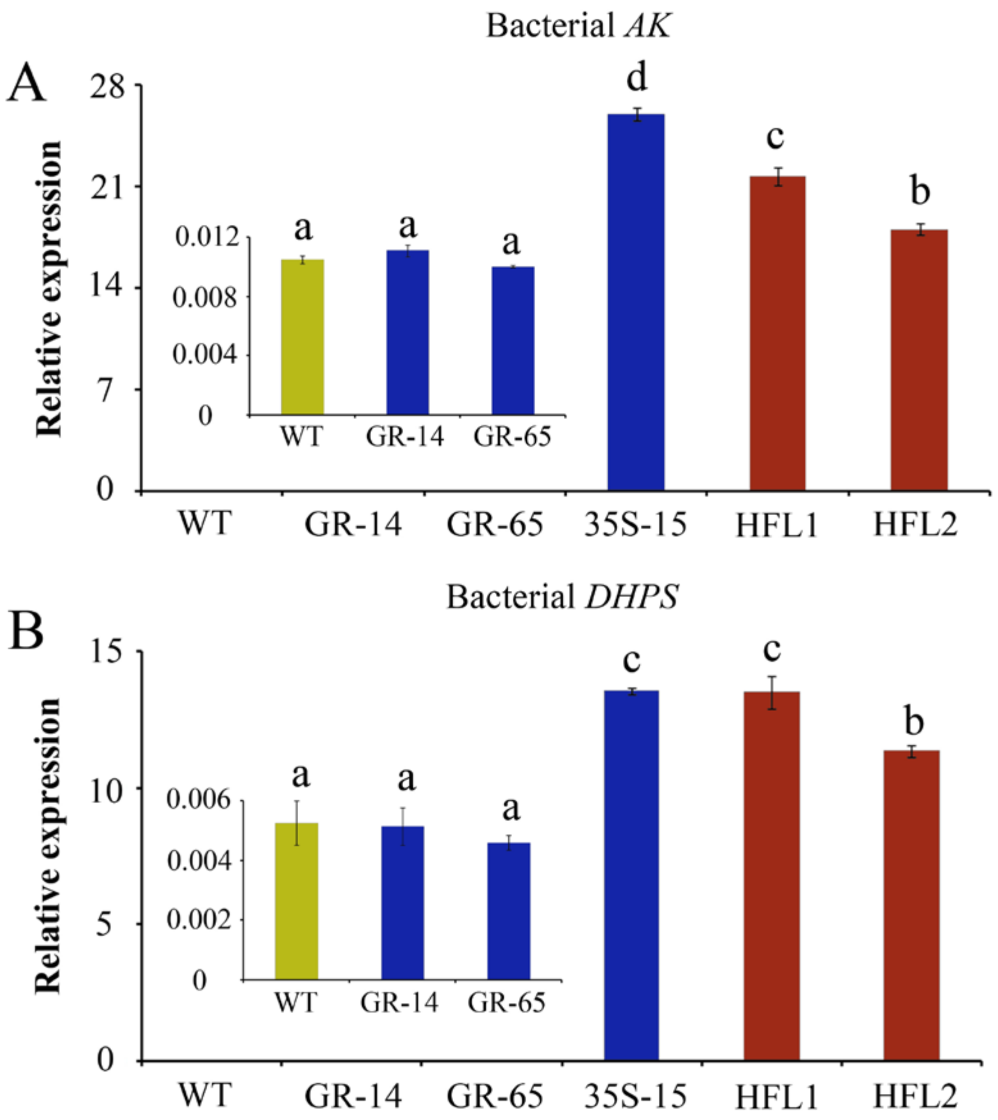

Supplementary Figure S2. Quantitative RT-PCR analysis of bacterial *AK* (A) and *DHPS* (B) transcript levels in leaves of wild-type and transgenic rice. *Actin* was used as the control. The error bars refer to SDs (standard deviation) of three biological replicates. The different letters indicate statistical significance among transgenic plants and wild type at $P<0.05$.

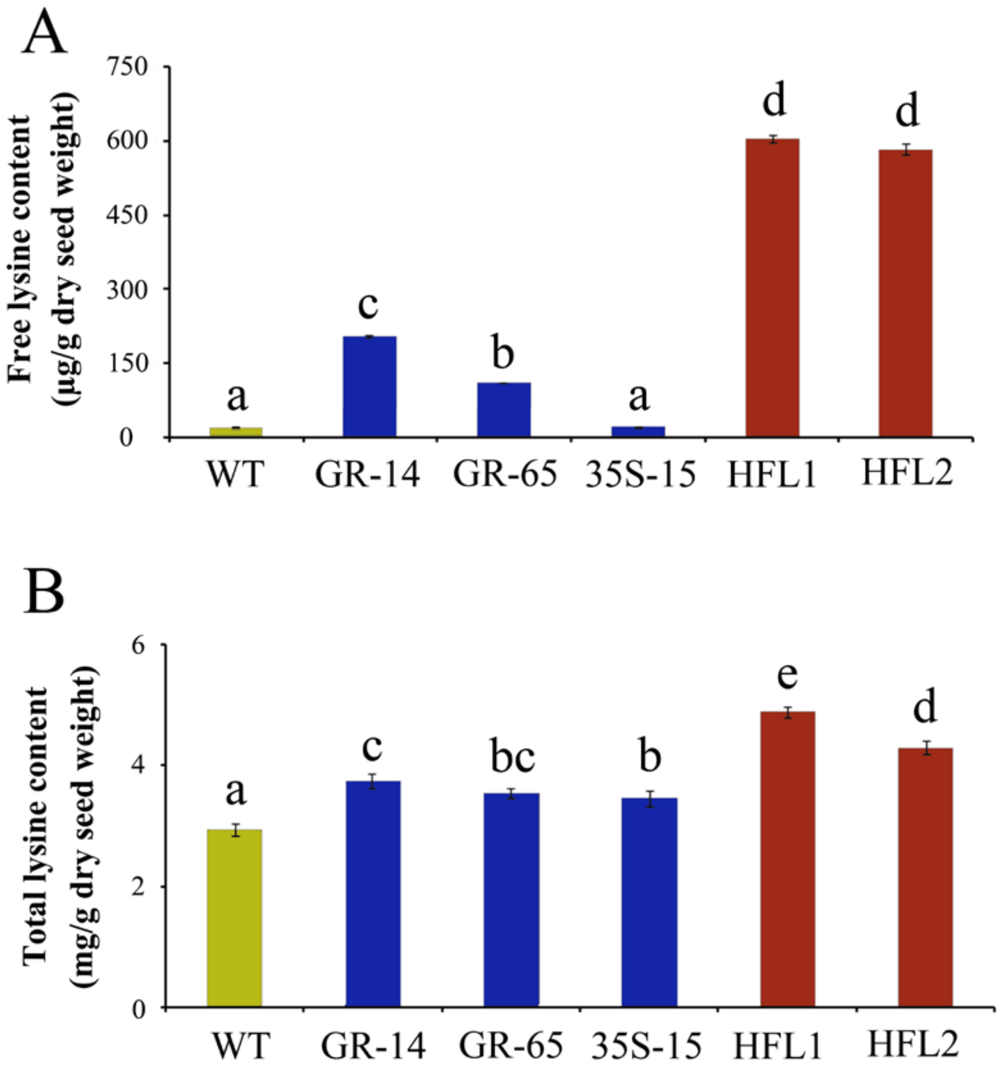

Supplementary Figure S3. The contents of free (A) and total lysine (B) in mature grains of transgenic and wild-type rice (Yangzhou, 2009). The error bars represent SDs of three biological replicates. The different letters indicate statistical significance among transgenic plants and wild type at $P<0.05$.

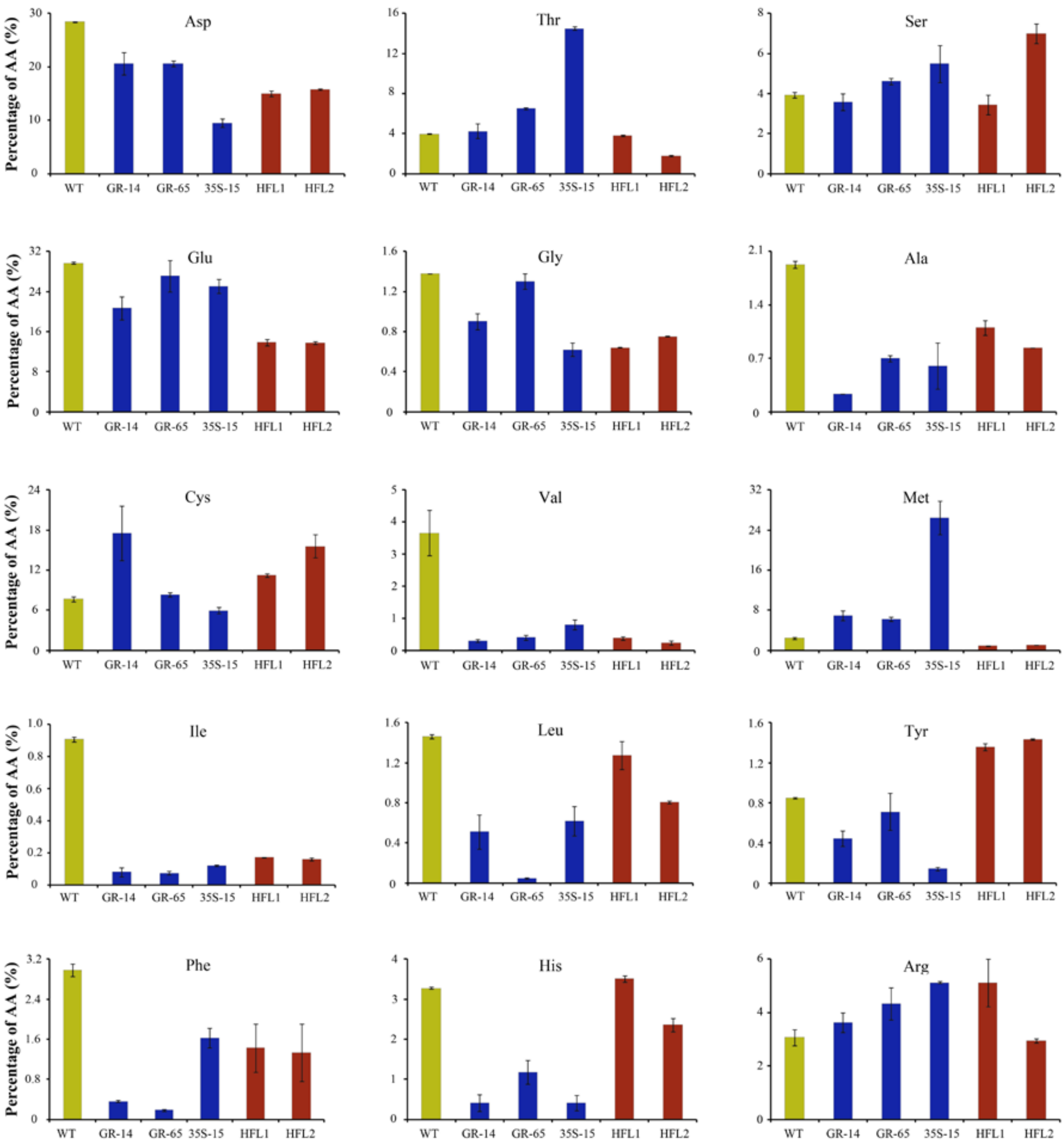

Supplementary Figure S4. Comparison of the percentage (by weight) of other individual free amino acids to total measurable free amino acids.

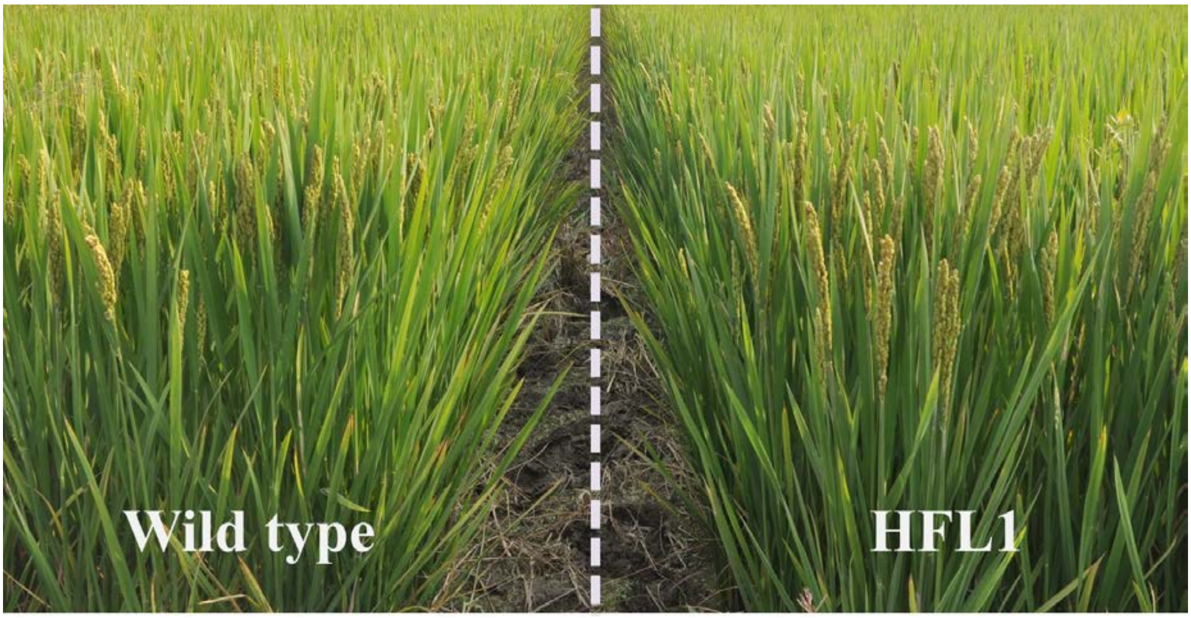

Supplementary Figure S5. Growth performance of wild type and HFL1 rice in the field.

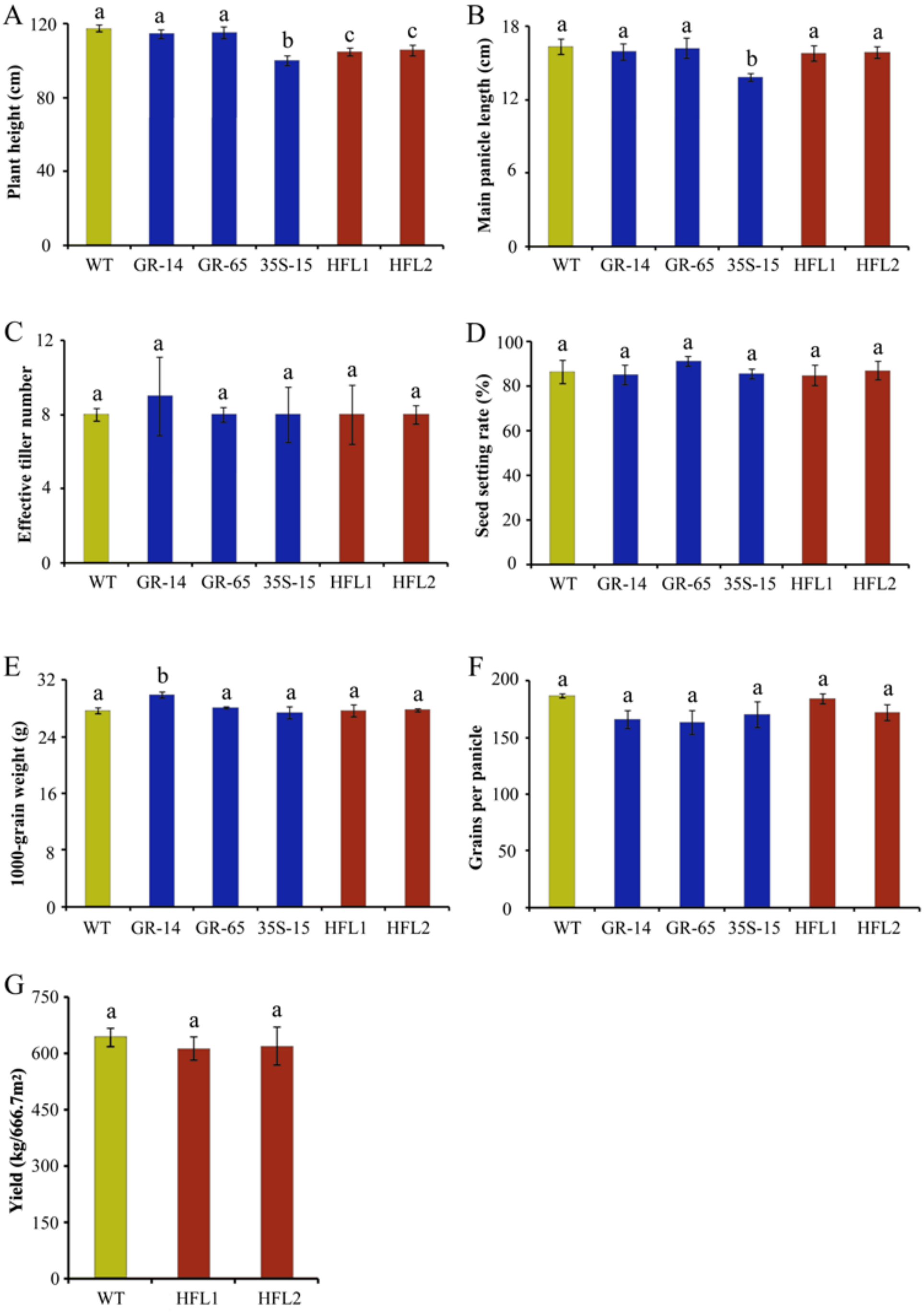

Supplementary Figure S6 The major agronomic traits of transgenic and wild-type plants (Yangzhou, 2013). Error bars are based on three independent biological replicates. The different letters indicate statistical significance among transgenic plants and wild type at $P<0.05$.

Supplementary Table S1. Primers used in this study.

Primer	Nucleotide sequence (5'-3')
P35s-F	TCCTTCGCAAGACCCTTCCTC
DHPS-2	TTACAGCAAACCGGCATG
LKR-R	CCTTAGCTGAGGCCAATCTAG
NOS-2	GACCGGCAACAGGATTCAAT
28-F3	GGAGCCCGTATGGTTGAACCATTTGGA
28-R3	TTGGTGTATGGAACTGTACGGGT
09-F	TTGCAGGGGTCTCTTTCG
09-R	TGCCTCTGGGTGGTTTTTG
10-F	TGTTTACTCCTTGCTTGATTGTT
10-R	TGGTAGTTTATGGAAGTCGTCTC
LB3	GTCCGCAATGTGTTATTAAGTTGTC
SP1	TTCTCCATAATAATGTGTGAGTAGT
SP2	ACCCTTAGTATGTATTTGTATTTG
SP3	GTCCGCAATGTGTTATTAAGTTGTC
SP4	TCTAGTAACATAGATGACACCGC
SP5	TAAATGTATAATTGCGGGACTC
SP6	GGACTCTAATCATAAAAACCCATC
Hyg-1	GCTGTTATGCGGCCATTGTC
Hyg-2	GACGTCTGTCGAGAAGTTTC
Akrt-F	CTGACACTTCGGACCACTGACTACC
Akrt-R	ACACCATCAACATCTTTCCATACCTG
DHSPrt-R	CCCTGCTCAGTTGCGTGAATAG
DHPSrt-F	GTAGTGAAGTGAAAAATCGGACATC
LKRrt-F	ACTCTTCAATGCTTGTAACATCTCC
LKRrt-R	AGTAGGGTTGCTTGGTGCTTT
EDHPS-RT1	TTCTTGCGACGGACGATTGTAGTAA
EDHPS-RT2	GATGAAAAAGGTAATGTCTGTCGGG
AK1-RT1	AGGAAGCACAGGTTATGGCTGGATG
AK1-RT2	AGTGAGCGTGGCATTAACCCTTGAT

Supplementary Table S2. Parameters of RVA profiles of flours and purified starches from mature rice grains.

	**Transgenic line**	**PKV***	**HPV***	**BDV***	**CPV***	**SBV***	**PeT***	**PaT***
Flour	WT	2916	1752	1164	3009	1257	6.2	71.1
	GR-14	2745	1645	1100	2951	1306	6.2	70.2
	GR-65	2749	1794	955	3077	1283	6.4	69.5
	35S-15	2483	1346	1137	2802	1456	6	71.9
	HFL1	2184	1140	1044	2538	1398	5.8	72.6
	HFL2	2097	1187	910	2451	1264	6.1	75.1
Starch	WT	884	792	92	1115	323	6.7	91.7
	GR-14	906	783	123	1120	337	6.9	92.4
	GR-65	908	789	119	1149	360	6.9	91.5
	35S-15	882	796	86	1218	422	6.7	91.6
	HFL1	804	728	76	1091	363	6.7	92.4
	HFL2	889	775	114	1078	303	6.9	91.7

* PKV, peak viscosity; HPV, hot paste viscosity; BDV, breakdown; CPV, cool paste viscosity; SBV, setback; PeT, peaking time; PaT, pasting temperature.
